# CD4^+^TGFβ^+^ cells infiltrated the bursa of Fabricius following IBDV infection, and correlated with a delayed viral clearance, but did not correlate with disease severity, or immunosuppression

**DOI:** 10.3389/fimmu.2023.1197746

**Published:** 2023-09-08

**Authors:** Salik Nazki, Vishwanatha R. A. P. Reddy, Nitin Kamble, Jean-Remy Sadeyen, Munir Iqbal, Shahriar Behboudi, Holly Shelton, Andrew J. Broadbent

**Affiliations:** ^1^ The Pirbright Institute, Woking, United Kingdom; ^2^ Nuffield Department of Medicine, Pandemic Sciences Institute, University of Oxford, Oxford, United Kingdom; ^3^ Department of Pathology and Infectious Disease, School of Veterinary Medicine, University of Surrey, Guildford, United Kingdom; ^4^ Department of Animal and Avian Sciences, University of Maryland, College Park, MD, United States

**Keywords:** Infectious bursal disease virus, IBDV, TGFβ, CD4^+^CD25^+^ T cells, regulatory T cells, bursa of Fabricius, immunosuppression

## Abstract

**Introduction:**

Infectious Bursal Disease Virus (IBDV) causes immunosuppression in chickens. While B-cell destruction is the main cause of humoral immunosuppression, bursal T cells from IBDV-infected birds have been reported to inhibit the mitogenic response of splenocytes, indicating that some T cell subsets in the infected bursa have immunomodulatory activities. CD4^+^CD25^+^TGFβ^+^ cells have been recently described in chickens that have immunoregulatory properties and play a role in the pathogenesis of Marek’s Disease Virus.

**Methods:**

To evaluate if CD4^+^CD25^+^TGFβ^+^ cells infiltrated the bursa of Fabricius (BF) following IBDV infection, and influenced the outcome of infection, birds were inoculated at either 2 days or 2 weeks of age with vaccine strain (228E), classic field strain (F52/70), or PBS (mock), and bursal cell populations were quantified by flow cytometry.

**Results:**

Both 228E and F52/70 led to atrophy of the BF, a significant reduction of Bu1^+^-B cells, and a significant increase in CD4^+^ and CD8α^+^ T cells in the BF, but only F52/70 caused suppression of immune responses to a test antigen in younger birds, and clinical signs in older birds. Virus was cleared from the BF more rapidly in younger birds than older birds. An infiltration of CD4^+^CD25^+^T cells into the BF, and elevated expression of bursal TGFβ-1^+^ mRNA was observed at all time points following infection, irrespective of the strain or age of the birds, but CD4^+^TGFβ^+^cells and CD4^+^CD25^+^TGFβ^+^ cells only appeared in the BF at 28 dpi in younger birds. In older birds, CD4^+^TGFβ^+^ cells and CD4^+^CD25^+^TGFβ^+^ cells were present at earlier time points, from 7dpi following 228E infection, and from 14 and 28 dpi following F52/70 infection, respectively.

**Discussion:**

Our data suggest that an earlier infiltration of CD4^+^TGFβ^+^ cells into the BF correlated with a delayed clearance of virus. However, the influx of CD4^+^TGFβ^+^ cells and CD4^+^CD25^+^TGFβ^+^ into the BF did not correlate with increased pathogenicity, or immunosuppression.

## Introduction

Infectious bursal disease virus (IBDV) is a member of *Birnaviridae* family which causes an economically important disease called infectious bursal disease (IBD) or Gumboro disease in poultry (1). Birds infected before 3 weeks of age usually exhibit subclinical infection with mild or no symptoms, but suffer long-lasting immunosuppression (2), whereas the clinical form of IBD is displayed in birds infected between 3-6 weeks of age (3). IBDV-mediated immunosuppression results in an increased risk of secondary infections and a reduced responsiveness to vaccines against other diseases (1), and the economic losses associated with the virus are due to both direct causes such as morbidity and mortality following infection, and indirect causes associated with immunosuppression. These economic consequences are vast enough to rank IBDV in the top five infectious problems of poultry (4, 5).

IBDV mainly replicates in IgM^+^ B lymphocytes in bursa of Fabricius (BF) of chickens, resulting in pathology and impaired function of the immune organ (3, 6), associated with suppression of humoral immunity (7, 8). Following infection, T cells infiltrate the BF, the majority of which are activated, and produce effector responses aimed at clearing infection (3). However, T cells from the BF of IBDV-infected birds have also been reported to inhibit the mitogenic response of splenocytes, indicating that some intrabursal T cell subsets have immunomodulatory activities following IBDV infection (3, 9). In mammals, regulatory T cells (Tregs) are a specialized subset of T cells which suppress or regulate other cells of the immune system to maintain homeostasis and self-tolerance (10, 11). Tregs induce suppression through the production of inhibitory cytokines such as interleukin-10 (IL-10) and transforming growth factor- beta (TGFβ) (12). In chickens, CD4^+^CD25^+^ T cells were initially proposed to have suppressive properties, similar to mammalian Tregs (13, 14), and were subsequently found to infiltrate the BF during IBDV infection (15), suggesting they could be responsible for the immunomodulatory activities described previously. However, as the activation of T cells may also lead to the upregulation of CD25, which itself is the IL-2 receptor α-chain, CD4^+^CD25^+^ T cells may not be true Tregs, and additional markers are needed to differentiate Tregs from other cell types in chickens. In mice, humans, pigs, and some other animals, Tregs are defined as the subpopulation of T cells which are CD4^+^CD25^+^FOXP3^+^ (16–19). While the FOXP3 gene has been identified in the chicken genome recently, monoclonal antibodies that target chicken FOXP3 and can be effectively used in flow cytometric assays are still lacking, so it is still not possible to define the chicken Treg population based on FOXP3^+^ expression at present (20). Recently, CD4^+^CD25^+^ T cells expressing surface TGFβ have been described in chickens that possess immune-regulatory properties (21). In this study, we hypothesized that some of the CD4^+^CD25^+^ T cells that infiltrated the BF following IBDV infection were also TGFβ^+^, and we tested this hypothesis by quantifying their number in the BF by flow cytometry at different time-points following IBDV infection, compared to mock-inoculated controls.

Moreover, in a recent study, a high and prolonged expression of IL-10 mRNA in the BF of layer-type birds compared to broiler-type birds correlated with a prolonged presence of CD4 cells, and a delayed recovery from infection, which the authors concluded could provide circumstantial evidence of the involvement of bursal Tregs in the recovery phase of IBDV (22). We hypothesized that the infiltration of CD4^+^TGFβ^+^ cells and CD4^+^CD25^+^TGFβ^+^ cells into the BF following IBDV infection may also delay the clearance of IBDV, which we tested by correlating the cell number in the BF at different time points with the amount of virus replicating in the bursa. Finally, as the CD4^+^CD25^+^TGFβ^+^ cells have previously been demonstrated to contribute to Marek’s Disease Virus (MDV) pathogenicity, we hypothesized that they may also play a role in determining the outcome of IBDV infection, and we tested this hypothesis by comparing their number in the BF between strains of differing virulence and immunosuppressive potential.

## Materials and methods

### Cells and viruses

The immortalized chicken B cell line, DT40 (23), was maintained in Roswell Park Memorial Institute (RPMI) media supplemented with sodium bicarbonate and L-glutamine (Sigma-Aldrich, Merck), 10% heat-inactivated (hi) fetal bovine serum (FBS), 1% tryptose phosphate broth (Sigma-Aldrich, Merck), 100 mM sodium pyruvate (Gibco), and 50 mM beta-mercaptoethanol (B-ME) (Gibco) (complete RPMI media) in an environment of 37°C and 5% CO_2_. A commercially licensed intermediate plus live vaccine (228E strain) and a stock of a classical (c)-strain, F52/70 (24), of IBDV (a kind gift from Dr Nicolas Eterradossi) (25) were used in subsequent experiments. Both the viruses were titrated using DT40 cells as previously described (26). The titre of the viruses was expressed as tissue-culture infectious dose_50_/mL (TCID_50_/mL) and calculated using the method described by Reed and Muench (27).

### Animal experiments

**Figure 1:**
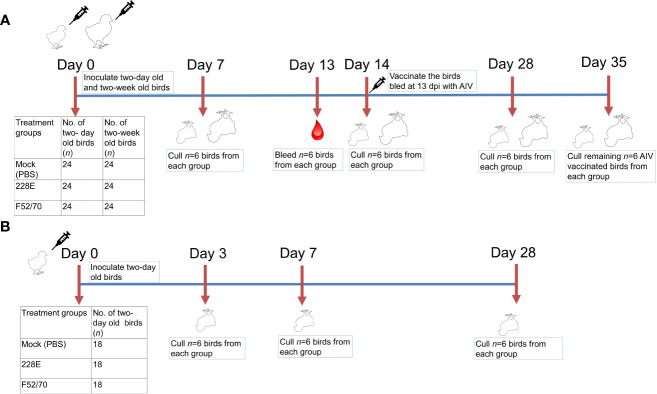
Overview of the animal studies. Two animal experiments were conducted in this study. In the first **(A)**, 72 two-day old and 72 two-week old Rhode Island Red chicken were randomly divided into three groups of 24 birds each and inoculated intranasally with PBS, 228E, or F52/70. Six chicken from each group were culled at 7, 14, 28 and 35 dpi to collect tissues and sera. At 13 dpi, six chicken from each group were bled and same birds were vaccinated subcutaneously with an inactivated AIV vaccine, and later culled at 35 dpi to check the level of immunosuppression due to IBDV in these birds. In the second animal experiment **(B)**, 54 two-day old chicken were divided into three groups of 18 birds each and inoculated intranasally with PBS, 228E, or F52/70. Six chickens from each group were culled at 3, 7, and 28 dpi to collect tissues. The body weight of each chicken was monitored daily, and the BF weight was observed at the day of cull.

In this study, two *in-vivo* animal experiments were conducted using specific pathogen free (SPF) Rhode Island Red (RIR) chickens of mixed sex, obtained from the National Avian Research Facility (NARF), Roslin Institute, University of Edinburgh (https://www.ed.ac.uk/roslin/national-avian-research-facility) and reared at The Pirbright Institute. In the first experiment, 72 two-day old chickens, and 72 two-week old chickens were randomly assigned into three groups of 24 birds (*n*=24) (**Figure 1A**). Each group was intranasaly inoculated with PBS (Mock), or 10^5^ TCID_50_/bird of vaccine strain 228E, or classical-strain F52/70, 50 μL per nares. Six birds from each group were humanely culled at 7, 14, 28 and 35 days-post-infection (dpi), or when the humane end point was reached. The BF was harvested from each bird at post-mortem for downstream analysis, weighed and stored in RNAlater (ThermoFisher Scientific) for RNA extraction. An additional six birds from each group were bled at 13 dpi to isolate peripheral blood mononuclear cells (PBMCs) to quantify immune cell populations. To determine whether IBDV infection led to immunosuppression, at 14 dpi, these six birds from each group were subcutaneously (s/c) inoculated a dose of 200 μL of 1024 haemagglutination (HA) units of inactivated avian influenza virus (AIV) vaccine, strain H9N2 (A/chicken/Pakistan/UDL-01/08) which was kindly provided by Dr. Munir Iqbal, The Pirbright Institute. Sera was collected from the birds at 28 days post IBDV inoculation (14 days post-inactivated AIV vaccine inoculation) to quantify anti-AIV antibody responses, and the birds were humanely culled at 35 dpi to collect the BF from each bird. The second experiment was conducted to confirm the results from the first study, and include an earlier time-point. Briefly, 54 two-day old chickens were inoculated, and six birds from each group were humanely culled at 3, 7, and 28 dpi, or when the humane end point was reached (**Figure 1B**). The BF and spleen were harvested from each bird at post-mortem for lymphocyte isolation and quantification by flow cytometry, and a part of the tissue was stored in RNAlater (ThermoFisher Scientific) for RNA extraction.

### Clinical scoring

Birds were weighed once daily and examined for clinical signs at least twice daily. A points-based scoring system, developed previously at The Pirbright Institute (25), was utilized to record the clinical scores as mild (score 1–7), moderate (score 8–11), or severe (score 12–17). Briefly, birds were scored on the basis of their behaviour with and without provocation, appearance, handling, and included an evaluation of the combs, wattles, feathers, crop, eyes and posture, as well as, breathing, interactions with the rest of the flock, and ability to evade capture (25). Sick birds were humanely culled on attaining the humane endpoints of 11 or above.

### RNA extraction, reverse transcription, and RT-qPCR

RNA was extracted from 25-30 mg of homogenized bursal tissue in RLT buffer, using the RNeasy kit (Qiagen) according to the manufacturer’s instructions. Complementary DNA (cDNA) was produced using SuperScript III Reverse Transcriptase (Invitrogen) and random primer. The reaction constituents and conditions were consistent with the manufacturer’s instructions. The cDNA was diluted 1:10, and virus was quantified by qPCR using the TaqMan™ Fast Universal PCR Master Mix (Applied Biosystems) according to manufacturer’s instructions. The primers and probes used for all the qPCR reactions are listed in **Table 1**, and qPCR amplification of the virus was achieved using a QuantStudio™ 5 qPCR machine (Applied Biosystems) with the following cycling conditions: 95°C for 1 min followed by 40 cycles of 95°C for 3 s, and 60°C for 30 s. The host cytokine genes were quantified by qPCR, using the Luna^®^ Universal qPCR mix (NEB) according to the manufacturer’s instructions, with a QuantStudio™ 5 machine, and the following cycling conditions: 95°C for 1 min, followed by 40 cycles of 95°C for 15 s and 60°C for 30 s, then a melt curve step at 95°C for 15 s, 60°C for 1 min, and 95°C for 15 s. The cycle threshold (CT) values were normalized to a housekeeping gene (RPLPO) and expressed relative to mock-inoculated controls in a ΔΔCT analysis.

**Table 1:** List of primers used in this study for RT-qPCR.

Target	Forward (5’ – 3’)	Reverse (5’ – 3’)	Probe or SYBR Green	Reference
IBDV	GAGGTGGCCGACCTCAACT	GCCCGGATTATGTCTTTGAAG	Probe: FAM-CCCCTGAAGATTGCAG GAGCATT-TAMRA	(25)
RPLP0	TTGGGCATCACCACAAAGATT	CCCACTTTGTCTCCGGTCTTAA	Probe: FAM-CATCACTCAGAATTTCAATGGTCCCTCGGG-TAMRA	(25)
TGF-β1	GTCCGGGCTCTGTACAACA	CCAATACTCATCGGGTCCAT	SYBR green	(28)
TGF-β2	ATCACCAGGACAGCGTTACA	CATCAAAAGACAACCATTCTCC	SYBR green	Designed
TGF-β3	GGAGGAGGAGAAGGAGGAGA	TAAAGCGGAACACATTGGAG	SYBR green	Designed
TGF-β Receptor 1	GGTTGGCAGTTAGGCATGAT	AACATCGACGAGCAATTTCC	SYBR green	(21)
IL10	GCTGAGGGTGAAGTTTGAGGAA	TGCTGATGACTGGTGCTGGT	SYBR green	(29)
IFN-γ	CAAAGCCGCACATCAAACA	TTTCACCTTCTTCACGCCATC	SYBR green	(29)

### Serology

The hemagglutination inhibition (HI) assay was conducted on sera collected at 28 days post IBDV-inoculation from birds vaccinated with an inactivated whole AIV vaccine comprised of strain UDL1/08 at 14 days post IBDV-inoculation. The HI assay was performed as described previously (30), using 1% chicken red blood cells.

### Single cell isolation

Peripheral blood mononuclear cells (PBMCs) were isolated from chicken blood samples by density gradient method using Falcon™ Centrifuge Tubes (Corning^®^Falcon) and Histopaque^®^-1083 Lymphocyte Separation Media (Sigma-Aldrich) according to the manufacturers’ instructions. The blood samples were layered onto Histopaque^®^-1083 solution at a ratio of 2:1 (blood:Histopaque) and centrifuged at 400 × g for 30 min without braking. The purified PBMC were collected, washed twice with sterile PBS (pH 7.0) and resuspended in 1 mL of FACS buffer (sterile PBS supplemented with 1% bovine serum albumin (BSA) (Sigma-Aldrich).

Mononuclear cells from the spleen were acquired by mashing the spleens through 40-mm-pore-size Falcon cell strainers (BD Bioscience) and overlaying the cells on Histopaque 1083 (Sigma-Aldrich) for density gradient centrifugation (400g for 30 min at 4°C). The buffy coat from the interface was collected, washed with sterile PBS (250g for 10 min at 4°C) and suspended in media containing RPMI-1640 media supplemented with 5% heat inactivated-foetal calf serum (HI-FCS) and penicillin-streptomycin solution.

The bursa of Fabricius (BF) samples were collected, washed with PBS, and transferred to a Petri dish containing 5 mL collagenase D solution (2.2 mg/mL, Sigma-Aldrich) in PBS. Using sterile scalpel blade, the BF samples were cut into smaller pieces and further incubated at 37°C for 30 min with periodic gentle agitation. Subsequently, the cell suspension was passed through 100 mm-pore-size Falcon cell strainers (BD Bioscience) and centrifuged (250g for 10 min at 4°C). The pellet was resuspended in RPMI-1640 media and, further, overlayed on Histopaque 1.083 (Sigma-Aldrich) for density gradient centrifugation (400g for 30 min at 4°C). The band of cells was collected from each sample, washed with sterile PBS, and suspended in media containing RPMI-1640 media supplemented with 5% heat inactivated-foetal calf serum (HI-FCS) and penicillin-streptomycin solution.

Finally, the mononuclear cells from the blood, spleen and BF were counted, and their viability was tested by trypan blue (Sigma-Aldrich) exclusion method (31).

### Flow cytometry

Mononuclear cells isolated from blood, spleen, and BF were stained with fluorochrome-conjugated monoclonal antibodies targeting chicken B cells (Bu1), CD4^+^ T cells, CD8^+^ T cells, and monocytes/macrophages (KUL01), and the expression of the surface molecules was quantified using flow cytometry. Briefly, after blocking Fc receptors with 4% BSA in PBS, the cells were incubated with anti-Bu1-FITC (Cambridge Bioscience), anti-KUL01-PE (Cambridge Bioscience), anti-CD4-PE/Cy7 (Cambridge Bioscience), anti-CD8α-Pacific Blue (Cambridge Bioscience), and Live/Dead™ fixable Near-IR dead cell stain (ThermoFisher) at 4°C for 15 min. The cells were washed twice with FACS buffer, fixed with 1% paraformaldehyde at 4°C for 15 min and finally resuspended in FACS buffer.

For quantifying the percentage of CD4^+^CD25^+^TGFβ^+^ cells, the previously established protocol (21) was followed, with slight modifications. In the first and second animal experiments, after blocking Fc receptors with 4% BSA in PBS, the cells were incubated with anti-CD4-PE (Cambridge Bioscience), anti-CD25-FITC mAbs (Bio-Rad), anti-TGF-β1,2,3-APC (R&D systems) and Live/Dead™ Fixable Aqua Dead Cell Stain (ThermoFisher) at 4°C for 15 min. The cells were washed twice with FACS buffer, fixed with 1% paraformaldehyde at 4°C for 15 min and finally resuspended in FACS buffer. In the second animal study, the antibody panel was modified to incorporate anti-CD8α mAb, and cells from each sample were also stained with a panel that included an isotype control for TGF-beta1,2,3-APC. Briefly, after blocking, the cells were incubated with anti-CD4-PE (Cambridge Bioscience), anti-CD8α-Pacific Blue (Cambridge Bioscience), anti-CD25-FITC (Bio-Rad), anti-TGF-beta1,2,3-APC (R&D systems) or isotype control, and Live/Dead™ fixable Near-IR dead cell stain (ThermoFisher) at 4°C for 15 min. The cells were washed twice with FACS buffer, fixed with 1% paraformaldehyde at 4°C for 15 min and finally resuspended in FACS buffer.

The suspension of the stained cell populations in FACS buffer was run on BD LSRFortessa™ Cell Analyzer (BD Biosciences). The data was analysed on FlowJo v10.8.1 software after setting compensation settings corresponding to monocolour and isotype control stains

### Data analysis

Graphical presentations and statistical analysis of the data were performed using GraphPad Prism 9.00 (GraphPad). Statistical calculation consisted of a one-way ANOVA for HI titre and immune cell analysis in PBMCs with Tukey’s multiple comparison test and Dunn’s multiple comparison test, respectively, while BF weight: Body weight (BF : BW) ratio, viral load, clinical score, immune cell analysis in tissues and cytokine expression was statistically compared by two-way ANOVA with a Tukey’s multiple comparison test and with a Bonferroni’s multiple comparisons test for viral load. Shapiro-Wilk normality test was used to check the normal distribution of data sets. Results were considered statistically significant if *p<0.05* and are indicated by asterisks significant (* p < 0.05, ** p < 0.01, *** p < 0.001, **** p < 0.0001).

## Results

### In older birds, IBDV was cleared more slowly than in younger birds, and strain F52/70 caused more substantial clinical signs than strain 228E. In younger birds, F52/70 caused significant suppression of immune responses to a test antigen, but 228E did not

**Figure 2:**
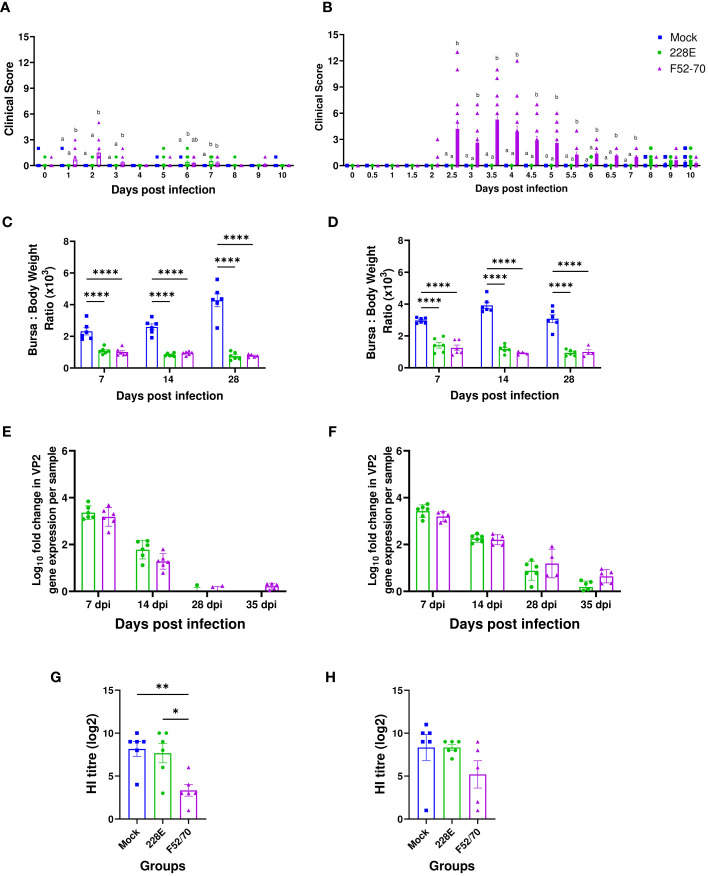
Effect of IBDV infection on clinical parameters and humoral immunity. **(A)** Two-day old and **(B)** two-week old birds were examined for clinical signs at least twice daily for first 10 days post infection and scored using a points-based scoring system developed previously at The Pirbright Institute as mild (1–7), moderate (8–11), or severe (12–17). Sick birds were humanely culled on attaining humane endpoints of 11 or above. **(C)** Two-day old and **(D)** two-week old birds were weighed every day in the morning and the BF weight was measured on the day of cull to determine the Bursa: Body weight ratio to compare the level of bursal atrophy between the mock and 228E and F52/70 infected birds. The viral loads in the BF obtained from mock and infected **(E)** two-day old and **(F)** two-week old birds were quantified by real-time reverse transcription quantitative PCR. The Log_10_ fold change in viral VP2 gene expression per sample was normalized to a housekeeping gene (RPLPO) and expressed relative to mock-inoculated controls in a ΔΔCT analysis. Sera was collected at 35 dpi from the **(G)** two-day old and **(H)** two-week old birds vaccinated with AIV vaccine at 14 dpi to observe the effect of IBDV on humoral immunity in the birds. A Hemagglutination inhibition (HI) assay was conducted on the sera using whole UDL1/08 influenza virus and 1% chicken red blood cells. The bars represent the means, and the error bars represent the standard errors of the mean (SEM). The different letters over the bars correspond to the values with a statistically significant difference from each other. Bars showing asterisks (*) represent values that differ significantly from each other (* indicates p ≤ 0.05, ** indicates p ≤ 0.01, and **** indicates p ≤ 0.0001).

Birds inoculated with 228E and F52/70 viruses at 2 days of age had either no signs, or mild clinical signs up to ten days post infection, with a maximum clinical score of 2 (strain 228E) or 5 (strain F52/70) (**Figure 2A**). Older birds inoculated with 228E at 2 weeks of age also had minimal clinical signs (maximum score of 2) consistent with its use as a vaccine, but older birds infected with F52/70 had substantial clinical signs, reaching a maximum score of 13 (**Figure 2B**). A significant difference in the BF weight: Body weight (BF : BW) ratio was observed between mock-inoculated and infected groups, regardless of strain or age (**Figures 2C, D**). The highest bursal viral load in 228E and F52/70 infected birds was at 7 dpi, which gradually decreased over the course of the study (**Figures 2E, F**). Virus was cleared from the BF of younger birds by 28 dpi, however, viral clearance from the BF of older birds was slower, with some bursal samples still PCR positive at 35dpi. There was no significant difference in the replication between the strains at any given time point within an age group. These data are consistent with IBDV being an acute infection, where replication peaks within a week of infection. To confirm immunosuppression in the birds, six birds from each group were inoculated subcutaneously (s/c) with an inactivated AIV vaccine, and sera collected at 28 dpi was tested for antibody responses against AIV using a hemagglutination inhibition (HI) assay. Younger birds infected with F52/70 were noted to be immunosuppressed as they exhibited a significantly lower HI titre (3.33), compared to 228E-inoculated (8.17) (p=0.0106) and mock-inoculated birds (7.67) (p=0.0048), respectively (**Figure 2G**). In older birds, there was a slight reduction in HI titre in F52/70 inoculated birds, but this did not reach statistical significance (**Figure 2H**). Taken together, both strain 228E and F52/70 caused bursal atrophy, but only F52/70 lead to a functional immunosuppression (in younger birds), and significant clinical signs (in older birds), and both strains were cleared at the same rate as each other, but clearance was slower in older birds than younger birds.

### IBDV infection led to changes in the immune cell populations in the BF

**Figure 3:**
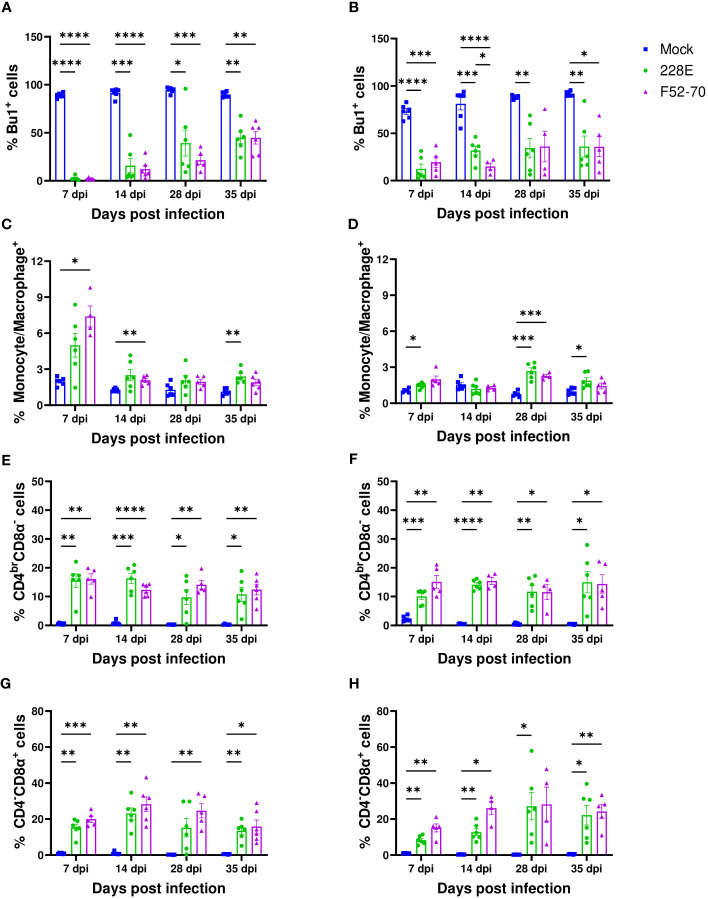
Quantification of the immune cell populations in the BF. Single-cell suspensions were immunostained to ascertain the frequencies of immune cell populations in BF collected from mock-inoculated birds, and birds inoculated with 228E and F52/70. The frequencies of Bu1^+^ cells in **(A)** two-day old and **(B)** two-week old, macrophages (Kul01^+^) in **(C)** two-day old and **(D)** two-week old, CD4^br^CD8α^-^ in **(E)** two-day old and **(F)** two-week old, and CD4^-^CD8α^+^ cell populations in **(G)** two-day old and **(H)** two-week old infected and uninfected birds at each time point are presented in the graphs. The Live/Dead-Fixable Near IR stain was used for dead cell exclusion. The bars represent the mean ± SEM for each population, and the asterisk (*) indicates a statistically significant difference between the averages found among three groups at each time point (* indicates p ≤ 0.05, ** indicates p ≤ 0.01, *** indicates p ≤ 0.001, and **** indicates p ≤ 0.0001).

To assess the effect of IBDV infection on the immune cell populations in the BF, we isolated cells from the BF of six birds from each group weekly, and conducted flow cytometric analysis. The gating strategy used is shown in **Supplementary Figure 1**. A statistically significant decline in Bu1^+^ cells was observed in the BF from IBDV-infected birds, compared to mock-inoculated birds, irrespective of strain or age (**Figures 3A, B**). Bu1^+^ cells started to repopulate the BF after 7 dpi but did not reach the original number, even after 35 dpi. An influx of monocytes/macrophages was observed in the acute stage of infection at 7 dpi in younger birds, which declined at later time-points, but this early influx was not detected in older birds (**Figures 3C, D**). There was also an influx of CD4^br^CD8α^-^ and CD4^-^CD8α^+^ cells into the BF of infected birds, compared to mock-inoculated birds, irrespective of age or strain. This influx was maintained for the duration of the experiment (**Figures 3E–H**). Overall, the IBDV infection in the BF destroyed the B cells, and led to an influx of CD4^+^ and CD8α^+^ cells.

### TGFβ mRNA expression remained elevated in the BF of IBDV infected birds 7-35dpi

**Figure 4:**
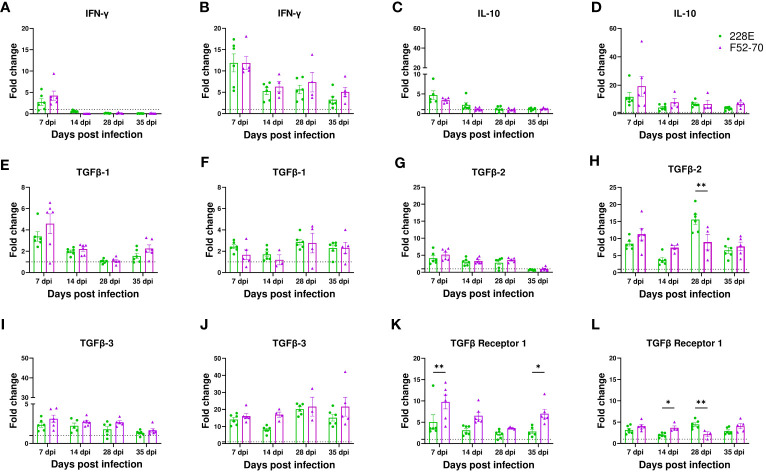
mRNA expression of cytokine genes in the BF. Analysis of mRNA transcription of IFN-γ in **(A)** two-day old and **(B)** two-week old, IL-10 in **(C)** two-day old and **(D)** two-week old, TGFβ-1 in **(E)** two-day old and **(F)** two-week old, TGFβ-2 in **(G)** two-day old and **(H)** two-week old, TGFβ-3 in **(I)** two-day old and **(J)** two-week old, and TGFβ receptor-1 in **(K)** two-day old and **(L)** two-week old in BF collected from infected and mock-inoculated birds was performed using RT-qPCR to compare the fold change in expression of the mRNA levels in infected birds compared to mock birds. Fold change was normalized the RPLPO housekeeping gene, and expressed relative to mock-inoculated birds in a in a ΔΔCT analysis. The bars correspond to the mean ± SEM for each population, and the asterisk (*) indicates a statistically significant difference between the groups (* indicates p ≤ 0.05, and ** indicates p ≤ 0.01).

Given that CD4 cells infiltrated the BF following IBDV infection, we quantified the expression levels of some of the cytokines that could be produced by these cells in the bursal samples by RTqPCR: IFN-γ, IL-10, and TGFβ-1. Gene expression was normalized to the house keeping gene RPLPO, and expressed relative to mock-inoculated birds, as fold change in mRNA levels. At 7 dpi, an increase in the mRNA expression of IFN-γ was observed in birds infected with IBDV when compared with mock-inoculated birds, implying that CD4 cells at this time point mounted an effector response aimed at clearing the infection. This increase was sustained in older birds, but declined in younger birds (**Figures 4A, B**), and there was no significant change between birds infected with strains 228E and F52/70. Similarly, the IL-10 mRNA levels were increased in IBDV infected birds at 7 dpi, which later reduced (**Figures 4C, D**). Interestingly, the expression of TGFβ-1 mRNA was increased following IBDV, in younger and older birds, and remained elevated throughout the study period from 7dpi to 35 dpi (**Figures 4E, F**). Based on this finding, we also quantified the level of expression of TGFβ-2, TGFβ-3, and TGFβ-receptor 1, and found that all were elevated in the BF of IBDV infected birds compared to mock-inoculated birds (**Figures 4G–L**).

### CD4^+^ TGFβ^+^ cells and CD4^+^CD25^+^ TGFβ^+^ cells infiltrated the BF of the IBDV infected birds, and their numbers varied with the age of bird and strain of virus.

**Figure 5:**
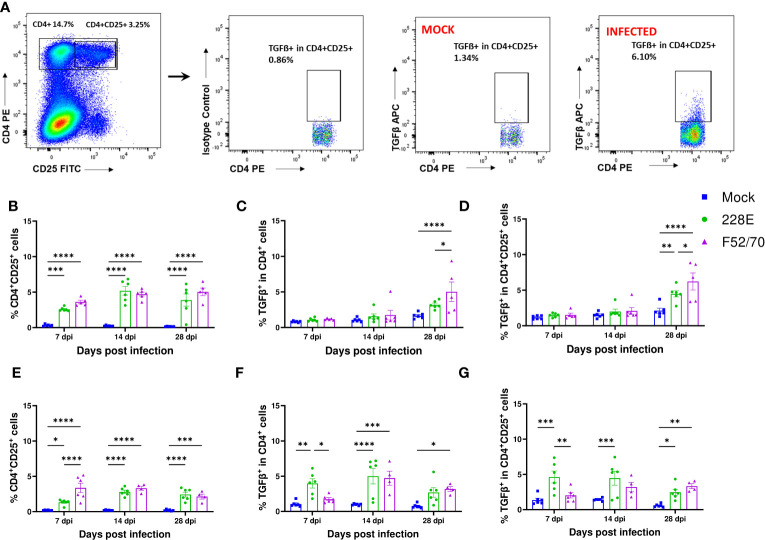
Identification of TGFβ producing cells in BF in IBDV infected birds. **(A)** The representative gating scheme for regulatory T cells in BF. Mononuclear cells were isolated from the BF of mock and infected birds and were stained with anti-CD4-PE, anti-CD25-FITC and anti-TGFβ-APC mAbs or isotype controls. The Live/Dead-Fixable aqua stain was used for dead cell exclusion. The percentages of **(B)** CD4^+^CD25^+^ T cells, **(C)** TGF-β^+^ in CD4^+^ T cells and **(D)** TGF-β^+^ in CD4^+^CD25^+^ cells in mock-inoculated and 228E and F52/70- infected two-day old birds and **(E)** CD4^+^CD25^+^ T cells, **(F)** TGF-β^+^ in CD4^+^ T cells and **(G)** TGF-β^+^ in CD4^+^CD25^+^ cells in two-week old birds are shown. The mean ± SEM value are shown as bars. * indicates a statistically significant difference (* indicates p ≤ 0.05, ** indicates p ≤ 0.01, *** indicates p ≤ 0.001, and **** indicates p ≤ 0.0001).

We quantified the number of bursal T cells that had membrane-bound TGFβ, by flow cytometry using the gating strategy described (**Figure 5A**). A significant increase in the number of CD4^+^CD25^+^ T cells was observed in the BF of IBDV infected birds, compared to the mock-inoculated birds at all time points from 7 dpi to 35 dpi, irrespective of strain or age (**Figures 5B, E**). However, a statistically significant increase in TGFβ^+^ expressing CD4^+^ cells and TGFβ^+^ expressing CD4^+^ CD25^+^cells was only observed at 28 dpi in younger birds (**Figures 5C, D**), when most of the virus had already been cleared from the BF, with significantly more CD4^+^CD25^+^ TGFβ^+^ cells in the BF of chickens infected with strain F52/70, compared to 228E (p=0.0304). In older birds, CD4^+^TGFβ^+^ cells and CD4^+^CD25^+^ TGFβ^+^ cells were present at earlier time points, from 7dpi following 228E infection, and from 14 and 28dpi following F52/70 infection, respectively (**Figures 5F, G**). Taken together, we were able to detect the presence of CD4^+^TGFβ^+^ cells and CD4^+^CD25^+^ TGFβ^+^ cells in the BF of IBDV-infected birds, which varied with the age of the birds and the strain of the virus.

### Quantification of immune-cell populations in different tissues suggests that the increase in T cells observed in the BF was the result of infiltration from elsewhere

**Figure 6:**
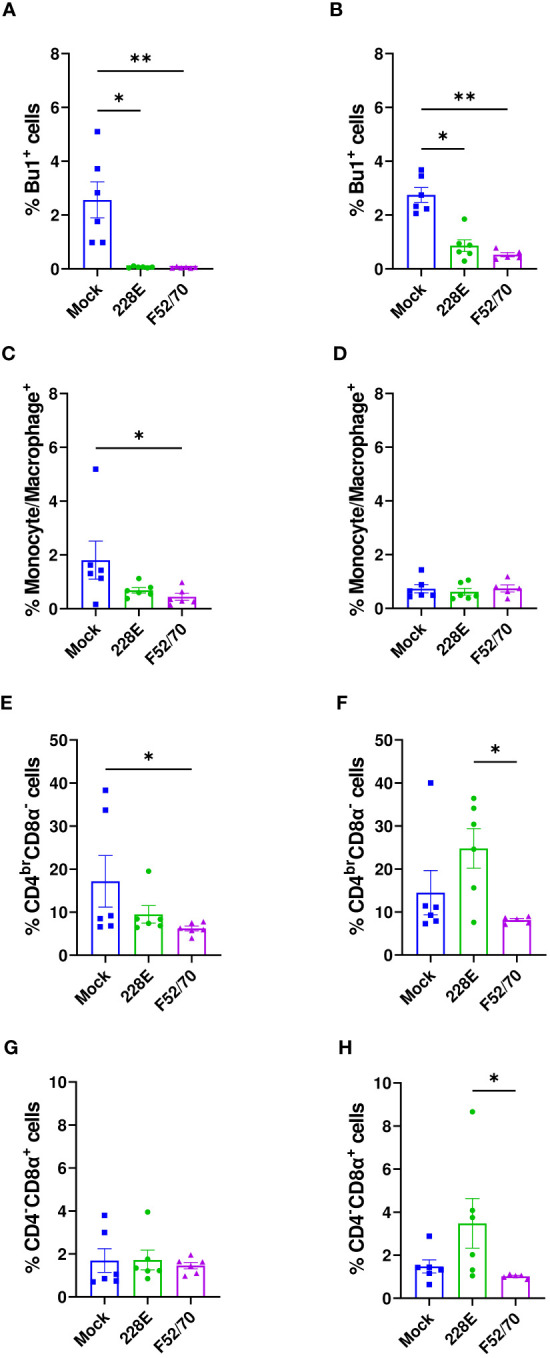
Quantification of the immune cell populations in the PBMCs. At 13 dpi, six birds from each group were bled to isolate PBMCs and stained for flow cytometric analysis. The frequencies of Bu1^+^ cells in **(A)** two-day old and **(B)** two-week old, macrophages (Kul01^+^) in **(C)** two-day old and **(D)** two-week old, CD4^br^CD8α^-^ in **(E)** two-day old and **(F)** two-week old, and CD4^-^CD8α^+^ cell populations in **(G)** two-day old and **(H)** two-week old infected and uninfected birds are presented in the graphs. The bars represent the means, and the error bars represent the standard errors of the mean (SEM). An asterisk (*) over bar indicates a significant difference (* indicates p ≤ 0.05, and ** indicates p ≤ 0.01).

**Figure 7:**
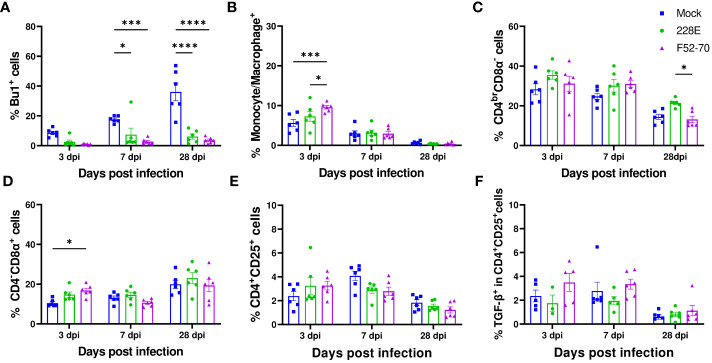
Quantification of the immune cell populations in the spleen. In the second animal experiment, splenocytes were isolated from the birds at 3, 7, and 28 dpi to detect the changes in the immune cell populations. In one panel, splenocytes were stained with anti-Bu1-FITC, anti-Chicken monocyte/macrophage-KUL01-PE, anti-CD4-PE/Cy7, and anti-CD8α-Pacific Blue. Another panel used anti-CD4-PE, anti-CD8α-Pacific Blue, anti-CD25-FITC, anti-TGF-β1,2,3-APC or isotype control to stain the splenocytes. Dead cells were excluded by using the Live/Dead-Fixable Near IR stain. Similar gating strategy as employed in BF was followed. The percentages of single positive **(A)** Bu1^+^ cells, **(B)** monocyte/macrophage^+^ (KUL01^+^), **(C)** CD4^br^CD8α^-^ and **(D)** CD4^-^CD8α^+^ are represented in the graphs at the indicated time points. The changes in the percentages of **(E)** CD4^+^CD25^+^ and **(F)** TGFβ^+^ in CD4^+^CD25^+^ cells were also determined. The frequencies are represented as the mean ± SEM value and shown as bars. * indicates a statistically significant difference (* indicates p ≤ 0.05, *** indicates p ≤ 0.001, and **** indicates p ≤ 0.0001).

We collected PBMCs from six birds in each group at 13 dpi, and quantified the percentages of B cells (Bu1^+^), monocytes (Kul01^+^), CD4^+^ and CD8α^+^ cells using flow cytometry, following the gating strategy depicted in **Supplementary Figure 1**. A significant reduction in the Bu1^+^ cells was observed in the PBMCs from birds infected with 228E and F52/70, compared to the mock-inoculated birds, irrespective of the age of the birds (**Figure 6**). B cells almost disappeared from the peripheral blood following IBDV infection, confirming the immunosuppressive state of the infected birds. In addition, in younger birds, there was a significant decrease in the percentage of monocytes and CD4^br^CD8α^-^ cell populations in the PBMCs from F52/70 -infected birds compared to mock birds, which could be the result of cellular efflux from the blood into infected tissues, such as the BF. As observed in the BF and PBMCs, there was also a decline in the Bu1^+^ cell population in the spleen (**Figure 7A**). In addition, although the percentage of monocytes/macrophages transiently increased in the spleen, and there was no significant difference at 7 or 28 dpi between the uninfected and infected groups (**Figure 7B**). Similarly, there was no prominent upregulation of CD4^br^CD8α^-^ and CD4^-^CD8α^+^ populations in the spleen at 7 or 28 dpi, compared to mock-inoculated controls, but there was a slight increase in CD4^-^CD8α^+^ cells in F52/70- infected birds compared to mock birds at 3 dpi (**Figures 7C, D**). There was also no significant difference in the populations of CD4^+^CD25^+^ cells, or CD4^+^CD25^+^TGFβ^+^ cells between the IBDV infected birds and the mock inoculated birds (**Figures 7E, F**). Taken together, the increase in the subsets of T cell populations we observed in the BF did not occur in the spleen, and there was a reduction in T cell numbers in the blood, suggesting that rather than there being a systemic increase in T cell production in multiple organs, the cells instead infiltrated the BF from elsewhere, due to the presence of replicating virus.

## Discussion

IBDV infection is known to cause a loss of bursal B cells and an increase in infiltrating T cells. The destruction of B cells is what drives IBDV-mediated immunosuppression, leading to a loss of humoral immunity, while T cell infiltrates predominantly are comprised of activated cells that produce effector responses aimed at clearing infection. However, T cells taken from the bursa following IBDV infection have been shown to inhibit the mitogenic responses of splenocytes, indicating that some infiltrating subsets have immunomodulatory properties (32). It is possible that these cells are Treg cells, which mediate regulatory activities through IL-10 and TGFβ (13). Previously, it was demonstrated that chicken CD4^+^CD25^+^ cells produce suppressive cytokines IL-10 and TGFβ, and may be immunomodulatory (13, 14), and it was subsequently found that they infiltrate the BF following IBDV infection (15). However, as CD25 is an activation marker, they may not represent true Treg cells (20) and additional markers are necessary to identify immunomodulatory cells. In the absence of adequate antibodies for identifying FOXP3 in chickens, CD4^+^CD25^+^TGFβ^+^ cells have recently been identified and described as immunomodulatory. We hypothesised that some of the CD4^+^CD25^+^ cells infiltrating the BF may be TGFβ^+^. We therefore set out to quantify their number in the BF by flow cytometry at different time-points following IBDV infection, compared to mock-inoculated controls.

Moreover, owing to circumstantial evidence that intrabursal Tregs may delay the recovery from IBDV infection (22), we sought to determine whether the infiltration of CD4^+^CD25^+^TGFβ^+^ cells into the BF following IBDV infection may also delay the clearance of IBDV, which we tested by correlating their cell number in the BF at different time points with the amount of virus replicating in the bursa. Finally, as the CD4^+^CD25^+^TGFβ^+^ cells have previously been demonstrated to contribute to Marek’s Disease Virus (MDV) pathogenicity, we compared their number in the BF between strains of differing virulence and immunosuppressive potential to evaluate whether they affected the outcome of IBDV infection. To this end, we quantified the numbers of different immune cell populations in the BF following infection with a vaccine strain (228E) or a virulent strain (F52/70) in birds inoculated at either 2 days of age, or 2 weeks of age, and we evaluated changes in bursal immune populations in the BF over the recovery phase of IBDV, from 7-35dpi.

It is known that after IBDV infection, there is an extensive loss of B cells in the BF, which then start repopulating the organ from 14 dpi (3, 33). In this study, we also observed significant bursal atrophy following IBDV infection, in birds infected with either vaccine strain 228E or classic field strain F52/70, and both strains led to a significant reduction in bursal B cell numbers initially (**Figures 2C, D**, and **3A, B**), which then recovered in number over time. This is consistent with strain F52/70 being a virulent field strain, and with 228E being an intermediate-hot vaccine, known to cause bursal lesions in its own right. Interestingly, despite these changes, only the classic field strain F52/70 resulted in statistically significant humoral immunosuppression in young birds, as evidenced by a reduction in the antibody response against an inactivated influenza vaccine given 14 days post-IBDV inoculation (**Figure 2G**), which suggests that even though IBDV vaccine strain 228E causes bursal lesions, this does not seem to impair immune responses against other vaccines. Moreover, F52/70 infection led to substantial clinical scores in older birds, whereas 228E did not. Taken together, the fact that F52/70 induced immunosuppression (in younger birds) and clinical disease (in older birds) whereas 228E did not enabled us to determine if the differences in disease outcome correlated with differences in immune cell populations in the BF.

Previous studies have reported a transient increase in monocytes/macrophages (34), and in this study, we also observed an increase in KUL01^+^ cells in the BF at the earliest time point we studied in younger birds (**Figure 3C**), consistent with inflammation following infection, which subsequently declined over the course of the experiments. Moreover, it is known that CD4^+^ and CD8^+^ T cells infiltrate the BF of IBDV infected birds and can be as high as 65% of the BF cell population (35). In this study, we also observed a substantial influx of CD4^+^ and CD8^+^ cells into the BF (**Figures 3E–H**), consistent with these previous reports. However, in our study, the T cell influx into the BF persisted throughout the course of the experiments. As we detected virus nucleic acid by RTqPCR until 28dpi in younger birds, and 35dpi in older birds, it is possible that the persistence of viral antigen in the BF drove the sustained T cell influx (36, 37). Further research is necessary to fully understand the reasons and implications of the persistence of T cells in BF after IBDV infection.

While IBDV-mediated immunosuppression is due to the destruction of B cells and the loss of humoral immunity, the clearance of IBDV, and recovery from infection, is known to be aided by bursal T-cell-mediated responses: For example, in one study, Cyclosporin A-induced T cell depletion resulted in an increase in bursal viral load (35), and in another study there was enhanced bursal mRNA transcript levels of cytokines IL-1b, IL-6, CXCLi2, and IFN-γ in IBDV-inoculated birds (38). In another study, upregulation of Th1 cytokines [IFN-γ, interleukins (IL)-2 and IL-12p40] and Th2 cytokines (IL-4, IL-5, IL-13 and IL-10) was thought to play a crucial role in driving cellular immune responses against IBDV in the acute phase of infection (39). Consistent with these data, in the present study, we observed an increase in IFN-γ mRNA expression in the BF of the IBDV infected birds at 7 dpi, that was sustained in older birds. However, in the present study, we also observed a sustained upregulation of the genes expressing the immunosuppressive cytokines TGFβ-1, 2 and 3, compared to mock controls (**Figure 4**). Previously, an upregulation of TGFβ2 expression was observed in PBMCs of chickens infected with IBDV, which was linked to limiting inflammation mediated by Th1 cytokines (40), and another two studies reported an increase of TGF-β mRNA expression in the BF of IBDV infected birds (22, 41), and it has been associated with an induction of bursal tissue fibrosis in the late stage of infection (41). TGFβ is produced or released by a range of immune cells, including T cells and monocytes/macrophages (42). In the current study, however, the source and role played by TGFβ was not clear. We observed the elevated levels of TGFβ in the BF of infected birds from 7 dpi until 28 dpi, even after KUL01^+^ cell populations were reduced to normal. These results led us to hypothesize that the prolonged elevation of TGFβ expression may be due to infiltrating T cells.

In mammals, Tregs produce both surface-bound and secreted forms of TGFβ (43), and in humans, TGFβ attaches to the surface of the Foxp3^+^Treg cell via the membrane anchoring molecule GARP (LRRC32). Cells that are positive for surface TGFβ are considered to be activated Tregs, which possess immune regulatory capabilities (43–46), and recent studies have also shown that surface bound TGFβ is involved in cell-contact dependent immunosuppression of Tregs (47, 48). Therefore, in the absence of adequate chicken Foxp3 flow cytometry markers, we quantified the number of CD4^+^ cells and CD4^+^CD25^+^ cells that also had surface TGFβ in the BF following IBDV infection. In order to increase scientific rigor, we included an isotype control for every sample.

In younger birds, CD4^+^TGFβ^+^ cells were only present in the BF from 28dpi, however, these cells infiltrated the BF earlier in older birds (present from 7dpi following 228E infection, and from 14dpi following F52/70 infection). The earlier presence of these cells in the BF correlated with a delayed clearance of virus, as in the younger birds, virus was cleared by 28dpi, whereas in older birds, virus nucleic acid could be detected even at 35dpi. Taken together, these data suggest that elevated CD4^+^TGFβ^+^ cells in the BF may delay the clearance of IBDV.

F52/70 produced more clinical signs than 228E in older birds, yet CD4^+^TGFβ^+^ and CD4^+^CD25^+^ TGFβ^+^ cells infiltrated the BF at earlier time points in 228E infected birds, indicating their earlier numbers in the BF did not correlate with increased disease severity. Previously, an upregulation of TGFβ2 expression was observed in PBMCs of chickens infected with IBDV, which was linked to limiting inflammation mediated by Th1 cytokines (40), so it is possible that early infiltration of these cells limits inflammation, although further studies are required to determine if this is the case. Moreover, F52/70 infection caused more immunosuppression in younger birds than older birds, and more immunosuppression than 228E in younger birds, however, the kinetics of CD4^+^CD25^+^ TGFβ^+^ cell infiltration was the same between younger and older birds infected with F52/70, and the kinetics of CD4^+^TGFβ^+^ and CD4^+^CD25^+^ TGFβ^+^ cell infiltration was the same between F52/70 and 228E infection in younger birds, suggesting the infiltration of these cells into the BF is unlikely to be contributing to immunosuppression.

To the best of our knowledge, this is the first study to that confirm the presence of CD4^+^TGFβ^+^ and CD4^+^CD25^+^TGFβ^+^ cells in the BF of chickens post-IBDV infection. While the infiltration of CD4^+^TGFβ^+^ cells into the BF was associated with a delayed viral clearance, our data suggest that neither CD4^+^TGFβ^+^ cells, nor CD4^+^CD25^+^TGFβ^+^ cells are likely to contribute to clinical disease severity, or immunosuppression. It is possible their role is to maintain homeostasis by suppressing unnecessary inflammatory and immune responses late in infection, or they could contribute to bursal fibrosis. Moreover, the source of the TGFβ in the BF at earlier time-points remains unknown, and the role and function of the CD4^+^CD25^+^cells that do not express TGFβ in the BF remains unknown, although these likely represent activated T cells. These are important questions to address in the future.

## Data availability statement

The original contributions presented in the study are included in the article/**Supplementary Material**. Further inquiries can be directed to the corresponding author.

## Ethics statement

All animal procedures were performed in accordance with the United Kingdom Animal (Scientific Procedures) Act (ASPA) 1986 under Home Office Establishment, Personal and Project licenses, following approval of the internal Animal Welfare and Ethic Review Board (AWERB) at The Pirbright Institute. The study was conducted in accordance with the local legislation and institutional requirements.

## Author contributions

SN performed experiments, analyzed data and wrote the first draft of the manuscript. VR, NK, and J-RS performed experiments and edited drafts of the manuscript. MI and SB supervised the study. SB edited the draft of the manuscript. HS and AB obtained funding. AB, HS, and SN designed the experiments. AB conceptualized the study, helped to conduct experiments and analyze data, and edited drafts of the manuscript. All authors contributed to the article and approved the submitted version.

## Supplementary material

Supplementary Figure 1 Gating strategy used to identify immune cell populations.
