# Supplementary figures and images for: CD4+TGFβ+ cells infiltrated the bursa of Fabricius following IBDV infection, and correlated with a delayed viral clearance, but did not correlate with disease severity, or immunosuppression
Source: Front Immunol. 2023 Sep 8;14:1197746. doi: 10.3389/fimmu.2023.1197746 (PMC10515216; doi:10.3389/fimmu.2023.1197746)

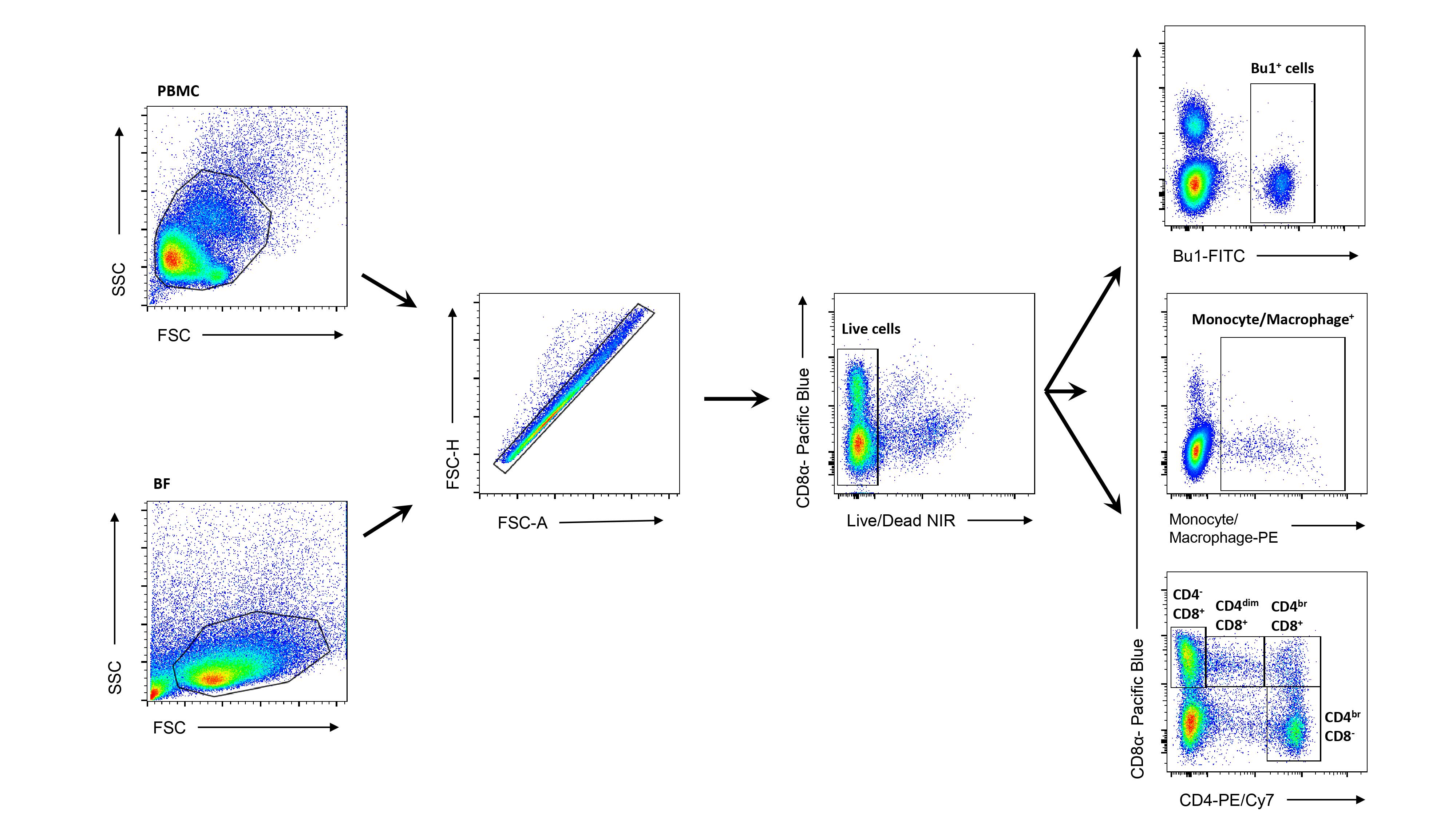

Supplement: Supplementary Figure 1 — Gating strategy used to identify immune cell populations. [file Image_1.jpeg]
